# Legacy Building from the Perspective of Palliative Care Professionals in Portugal: A Qualitative Thematic Analysis

**DOI:** 10.3390/nursrep15100366

**Published:** 2025-10-15

**Authors:** Carlos Laranjeira, Andréa Marques, Ana Fátima Fernandes, Maria Aparecida Domingos, Isabel Borges Moreira

**Affiliations:** 1 School of Health Sciences, Polytechnic University of Leiria, Campus 2, Morro do Lena, Alto do Vieiro, Apartado 4137, 2411-901 Leiria, Portugal; 2 Centre for Innovative Care and Health Technology (ciTechCare), Polytechnic University of Leiria, Campus 5, Rua das Olhalvas, 2414-016 Leiria, Portugal; 3 Comprehensive Health Research Centre (CHRC), University of Évora, 7000-801 Évora, Portugal; 4 Health Sciences Research Unit: Nursing, Nursing School of University of Coimbra, 3000-075 Coimbra, Portugal; 5 Department of Nursing, Federal University of Ceará, Fortaleza 60430-160, Brazil

**Keywords:** legacy, dignity-conserving care, palliative care, end of life, healthcare professionals, nursing

## Abstract

**Background/Objectives**: Legacy planning should respect te care preferences of people facing serious illness and integrate palliative care (PC). Legacy creation with the guidance of health professionals in PC assumes a therapeutic nature and aims to respond to the psychospiritual needs of patients and their families. To date, research on professional experiences to create legacy in PC remains scarce. Therefore, this study sought to explore the experiences of PC professionals in legacy creation with the ill person and their family during EoL care. **Methods**: A descriptive qualitative study was performed through in-person semi-structured interviews with PC professionals from Portugal. Data collection was conducted from February to May 2025. Transcripts from the interviews were thematically analyzed with qualitative data management software WebQDA. The study adhered to the Standards for Reporting Qualitative Research (SRQR) guidelines. **Results**: Sixteen PC professionals participated in the study. Most participants were nurses (n = 8), followed by six physicians and two psychologists. The mean age of participants was 44.93 ± 10.46 years. Data analysis yielded three themes: (1) the worth of legacy in EoL; (2) enablers of legacy-building process; and (3) challenges of legacy-building process. **Conclusions**: Legacy is a meaningful resource that gives professionals the opportunity to connect with patients and their families, and to enact value-concordant person-centered care. By providing a greater grasp of legacy construction, our findings may help healthcare providers better understand how to provide dying patients and their families with dignity-conserving care.

## 1. Introduction

Noncommunicable diseases (NCDs) such as cardio-respiratory diseases, cancers, and degenerative diseases remain the leading causes of death and disability in the world, including in the WHO European Region [1]. According to the WHO [2], annually, approximately 56.8 million individuals globally (25.7 million in their final year of life) require palliative care (PC). Many of these life-limiting conditions require PC because they produce physical, psychological, social, and spiritual suffering, especially in their end of life (EoL) stages. Upon receiving a terminal diagnosis, the individual becomes aware of their imminent mortality. This may induce a psychological disturbance leading to shock, thus modifying the individual’s perception of security [3]. Individuals alter their customary conduct as they strive to comprehend and ascribe significance to their conditions. In this sense, they contemplate their identity and lifestyle decisions, while examining life assumptions pertaining to the future, relationships, and autonomy [4,5]. A life review can reestablish chronological coherence and significance, but may also reveal unsolved matters [6].

There are differing assumptions regarding whether individuals react to a terminal diagnosis with similar coping mechanisms or if individual differences are more pronounced [7]. However, there is a consensus that most people endeavor to preserve control and normalcy [8]. In this vein, they establish objectives and engage in the resolution of pending matters. Legacies enable the partial collapse of ego barriers and create posthumous identities [9,10]. Discovering meaning during the illness trajectory and dying process alleviates suffering, because ill people often endure some form of loss and grief [11,12]. A predominant source of suffering throughout the dying process is the separation from loved ones [13]. Individuals express fear concerning the repercussions of their demise on others, particularly the welfare of dependent children. Profound anguish arises when death subverts all that holds significance for the individual. This may encompass physical alterations, apprehension with imposing on others, and the loss of control, duties, or goals [14]. The demoralization syndrome occurs when individuals encounter feelings of hopelessness, meaninglessness, and reduced self-esteem [15,16]. Therefore, upon cessation of curative treatment, it is essential to manage patients’ existential concerns. A variety of psychospiritual interventions may be employed to alleviate suffering and reestablish an individual’s connections with significant facets of life [17,18]. These include meditation, dignity therapy, life review, psychotherapy, and therapeutic touch that aim to facilitate quality of life, and emotional and spiritual well-being [18,19]. Other strategies such as active listening and presence help patients and relatives feel that professionals were indeed concerned about them during an existential crisis, inducing a sense of relief [17].

The Lancet Commission on the Value of Death reminds us that death and life are bound together, which means that without death there would be no life [20]. With this assumption, PC is founded on a humanistic, life-affirming philosophy that honors individuals for their history, memories, and life narratives, as well as their current condition and prospective achievements [21]. PC also offers a support system to help the family cope during the patient’s illness and in their own bereavement [21]. Key PC pillars include pain and symptom management, emotional and psychological support, spiritual support, social support and communication, and caregiver support [21]. This comprehensive care upholds an individual’s dignity by ensuring comfort, alleviating existential anguish, and satisfying their diverse anticipated needs, encompassing physical, psychospiritual, social, and environmental dimensions [22]. To achieve that purpose, a legacy offers a form of symbolic immortality, fulfilling a fundamental human drive to persist beyond physical death. Consequently, PC teams must implement activities that establish a ‘legacy’ for patients, as this approach aligns with the foundational philosophical principle of PC proposed by Cicely Saunders: “You matter because you are you, and you matter to the last moment of your life” [23]. Furthermore, Harvey Chochinov claimed that “intensive caring”, which describes a compassionate approach to holding or containing hope, enables healthcare practitioners to assist ill people endure suffering and help them feel that they matter [24]. Hence, legacy creation with the guidance of health professionals in PC assumes a therapeutic nature and aims to respond to the psychospiritual needs of patients and their families.

Timóteo et al. [25] theorized that “legacy serves as a unifying element that connects the past, present, and future, providing a sense of purpose in life. It implies the idea of having a life witnessed by others, whether planned or serendipitous. The goal is to generate memories or symbols/objects specifically crafted to elicit memories that will serve as a favorable encounter in the present and elicit positive emotions when recalled in the future”. Moreover, for Figueroa Gray et al. [26], legacy is seen “as a process (e.g., specific actions taken to create objects, experiences, or memories that could remain after death), a wish (e.g., the desire to leave something behind after death or influence others’ memories) or, less often, as a material object (e.g., the set of items left behind)”.

Building a legacy ensures that experiences can be shared and preserved for the future and helps people move toward self-transcendence, fostering awareness of their mortality [27]. Evidence suggests that legacy-building artifacts, including the creation of symbols or memories of oneself for future memory, can improve the adjustment processes of the ill person, family and health professionals in terms of well-being, grieving process and professional satisfaction, respectively [25,28,29]. Legacy creation favors a life review, pushing an individual’s self-boundaries through introspection and thus helping them reflect on its meaning, remember moments of happiness, improve social relationships, reconcile conflicts and regrets, enhance their sense of dignity, and prepare for the end of life (EoL) in a peaceful manner [26,30,31,32,33,34,35]. In this approach, the person is invited to talk about the life issues that are most important to them and that they wish to convey to those they love most. Writing a letter, making a drawing, or creating videos are simple forms of legacy, through which the person communicates experiences, virtues, wisdom and future advice. Other forms of legacy are, for example, the creation of a photo album, an autobiography, or the creation of an object to give as a gift or a box with significant objects [26]. Beyond material forms, Neller et al. [36] highlights the need to promote ethical will creation, which are ways of intangible legacy and non-legal expression to convey values to others and facilitate EoL preparation.

Previous studies indicate the existence of three broad groups of legacies: primary, secondary, and tertiary [33]. The primary legacy consists of tangible and social artifacts, including financial and legal documents, professional items, and keepsakes, which an individual intentionally bequeaths, along with their wishes for remembrance [34]. For people afflicted with serious illness, legacy planning may function as a rite of passage or a mechanism for adapting. It may also entail making decisions concerning medical treatment [30]. Secondary legacy refers to the grief and memorialization actions conducted by others in honor of a deceased loved one or family member [35]. Finally, tertiary legacy pertains to recognizing the professional, political, or worldwide impact of a public personality with whom the remembered may not have had a personal acquaintance [30].

Although valued by people at the end of their lives, dignity-conserving interventions are still incipient in PC practice [31]. Legacy creation—encompassing aspirations for posthumous remembrance and influence on others—represents an overlooked yet significant aspect of decision-making for individuals facing life-threatening illnesses [25,26]. Likewise, there are no known qualitative studies related to legacy-making in palliative care conducted in the Portuguese context. Therefore, the aim of this study was to explore the experiences of PC professionals in building legacy with the ill person and their family during EoL care. The research question was: What practices do PC professionals use when constructing a legacy with the person at the end of their lives and their family? We hope this study gives visibility to the construction of a legacy as a strategy to preserve dignity in PC and thus contributes to the consolidation of the paradigm of person-centered PC.

## 2. Materials and Methods

### 2.1. Study

Given the nature of the research question, a descriptive qualitative study was undertaken to facilitate a comprehensive knowledge of the perspectives and experiences of PC professionals in developing legacy initiatives. The research was grounded on the philosophical framework of critical realism, which is particularly effective for comprehending the mechanisms and reasons behind phenomena, as well as elucidating the impact of context on outcomes in a natural environment [37]. Critical realism lies between the positivist and interpretivist approaches, showing that knowledge production will always be fallible and subjective [37]. This research focused on individual decisions and actions regarding modifications in PC and support networks, facilitated by a critical realism approach.

The Standards for Reporting Qualitative Research (SRQR) criteria were followed to guarantee enough detail in reporting [38] (Supplementary Materials).

### 2.2. Setting, Participants and Recruitment

Participants were purposively recruited from specialized PC teams (including inpatient services, intra-hospital PC teams and home-based care services) that care for adults. Based on the EAPC Atlas of Palliative Care in the European region 2025 [39], 150 specialized services operate in Portugal (including islands) with a greater number of services in the Northern Region and Lisbon area (up to 97 teams), 24 in the Central Region, 12 in Alentejo, 7 in Algarve, 6 in Azores and 3 in Madeira. These PC teams are multidisciplinary, whose core members include nurses, physicians, psychologists, and social workers. Only a few teams include physiotherapists and occupational therapists.

To access potential participants, the Portuguese National Association for Palliative Care was contacted to disseminate the study through the mailing list of its associate teams. Professionals from five specialized PC teams in the northern and central regions of Portugal responded positively to the invitation. Recruitment began with sending the head of each PC team detailed information about the study. Parties would contact the principal investigator if they were still interested in participating. Then, the objectives of the study were presented, questions were clarified, and the informed consent form was made available by email. After 48 h, all participants were contacted by telephone to schedule the interview.

A maximum variation sampling technique was employed to attain optimal heterogeneity regarding gender, age, and seniority. The inclusion criteria comprised: (1) health professionals working in specialist PC teams (specifically nurses, physicians, and psychologists); (2) advanced training in PC; (3) at least 6 months of professional experience in PC; and 4) direct activity in caring for people at the EoL. Exclusion criteria were as follows: (1) professionals away from work due to vacation and medical leave; and (2) professionals assuming exclusively management positions.

### 2.3. Data Collection

Data were collected through semi-structured individual interviews conducted between February and May 2025, in a location convenient for the interviewees; all chose their place of employment. Only two interviews per day were scheduled to avoid disruptions to team activities. All interviews were conducted as ‘face-to-face’ conversations by the first author (C.L.), a male white registered nurse with a professional background in oncology care and experience in methods of qualitative data collection. The interviewer had no caring relationship or other connections with the participants.

The semi-structured interview guide was developed based on findings from previous research [10,40,41] and the clinical and research experience of the authors. The interview script consisted of two parts. The first part collects personal and professional variables to characterize the participants. The second part presents the interview questions, grouped into three domains: (1) meanings attributed to the construction of the EoL legacy; (2) experiences in the construction of the legacy; and (3) facilitating and hindering factors in its construction (Appendix A).

Open-ended questions were employed to prompt participants to discuss legacy in the context of their roles as PC professionals. Examples of inquiries included: “What does legacy mean to you?”; “What strategies can be employed to foster legacy in EoL?”; “What enablers and challenges do you face in implementing legacy-making interventions?”. Follow-up and probing inquiries were employed to get comprehensive data, such as, “Can you elucidate what you mean by that?” and “Could you provide an example?”. The script was verified by two experts (A.M. and I.B.M.) with experience on the subject, who made slight changes to the phrasing of the questions. The interviews ranged from 40 to 75 min and were audio recorded and transcribed. Audio recordings were made with an MP3 recorder, and pen and field notebook, with the consent of each participant.

A courteous and non-confrontational approach was employed during the interviews to mitigate social desirability and response biases. Strategies like open-ended inquiries, paraphrasing, reformulation, synthesis, and intervals of silence were employed to encourage participants to articulate how they felt. In addition, we aimed to establish suitable rapport by employing active listening, exhibiting empathy, and fostering a relationship based on trust. Each participant was interviewed one time. Furthermore, all transcripts were sent to participants for feedback and/or amendments.

Data collection and data analysis happened concurrently (i.e., after each interview, the researcher immediately analyzed the information). The conceptual scope of themes and codes became saturated following the 13th participant; however, three further participants were called for interviews as they had previously consented but were unavailable (prolonged engagement). All interviews were done in Portuguese. The quotations were initially translated into English and subsequently back-translated to Portuguese to ensure accurate translation.

### 2.4. Data Analysis

Reflexive thematic analysis proposed by Braun and Clarke [42] was used as the method for data analysis due to its capacity to elucidate how individuals interpret their surroundings, which is pertinent for examining subjective views. This approach is an inductive method that facilitates the establishment of emergent codes (individual concepts linked to a segment of data) and themes (patterns of collective significance) based on the original data. After each interview, data was transcribed verbatim and analyzed by the primary author (C.L.), who regularly engaged in discussions with the co-authors.

Braun and Clarke’s [42,43] six phases were followed: Phase 1 (Familiarization with data), the complete transcribed data corpus was meticulously read and re-read with an inquisitive mindset and a writing instrument in hand to achieve a comprehensive grasp of the entire material; Phase 2 (Code Generation), concise labels encapsulating pertinent meaning units were developed and improved. Data that was clearly irrelevant to the research questions remained uncoded, while consideration of both semantic and latent meanings resulted in certain data segments being double-coded; Phase 3 (Searching for potential themes), codes were categorized based on the identification of central organizing notions. Preliminary thematic maps were created to organize possible themes and determine their interrelations; Phase 4 (Reviewing themes), the proposed method of arranging material was evaluated and amended. Themes must be exhaustive and distinct, encapsulating pertinent meanings within the material; Phase 5 (Defining and naming themes), themes were assigned tentative names that are both evocative and descriptive, accompanied by a brief presentation; Phase 6 (Producing the report), the Findings section was formulated. While Braun and Clarke advocate for a “analytical” reporting style that integrates the findings and discussion sections [42], our study adhered to a consensus reporting approach and reserved the analytical component that extends beyond the dataset for a separate Discussion section.

Data storage and management were assisted by the qualitative data analysis software WebQDA (Version 3.0, University of Aveiro, Aveiro, Portugal).

### 2.5. Rigor

To ensure the rigor of the research, we employed four criteria to establish trustworthiness [44]: (i) credibility was established by ongoing research team meetings and dialogs with PC peers to deliberate on themes that surfaced from the data until a unanimous consensus was reached (A.F.F. and M.A.D.). The study was supervised by two nursing faculty members with expertise in human responses to chronic conditions, as well as in qualitative research methodologies (A.M. and I.B.M.). The same researcher conducted all interviews to establish a rapport of trust with each subject. Peer debriefing was performed to verify credibility and reliability. Reflexivity was preserved using field journals and writing memos to deliberate on the ongoing reflection about the research process; (ii) dependability was ensured by applying qualitative methodologies, which offer a rigorous and transparent strategy for data analysis. Purposive sampling was employed to achieve a diversified sample with different backgrounds; (iii) confirmability was demonstrated through participant validation; segments of the participants’ verbatim accounts are delineated here to illustrate the extracted themes concretely. Ultimately, (iv) transferability was established by offering an in-depth comprehension of PC experts’ experiences in managing legacy-making interventions to enable others to compare and critique the findings.

### 2.6. Ethics

The study was approved by Ethics Committee of Polytechnic University of Leiria, Portugal (CE/IPLEIRIA/26/2025 approved on 20 January 2025). In compliance with the ethical standards outlined in the Declaration of Helsinki [45], participants were provided with both oral and written information regarding the study’s objective, the voluntary nature of their involvement, their right to withdraw at any time without justification, and the confidentiality of the study. All participants provided written informed consent for their interviews to be audio-recorded and for data to be used in this study. No financial incentives were provided. Recordings and transcripts were designated an alphanumeric code to maintain data confidentiality (P1, P2, …).

## 3. Results

### 3.1. Participants

The sample size of sixteen interviewees comprised eight nurses, six physicians and two psychologists (Table 1). Most participants were female with a mean age of 44.93 ± 10.46 years (range: 28–59). Most participants worked in in-hospital palliative support teams (n = 6) and had a mean number of years of professional experience in PC of 6.60 ± 3.94 years. All professionals have a master’s degree or professional specialization in PC.

### 3.2. Overview of Findings

Thematic analysis of transcripts uncovered insights and recommendations from PC professionals across three overarching themes: (1) the worth of legacy in EoL; (2) enablers of legacy-building process; and (3) challenges of legacy-building process (Table 2).

#### 3.2.1. The Worth of Legacy in EoL

Many participants highlighted how legacy activities may significantly contribute to (a) a process of memory-making (Memorabilia), (b) an enduring connection in the context of anticipated death, and (c) seeking validation for one’s existence.

(a) Process of memory-making—Memorabilia

Participants discussed the act of creating memorabilia—legacy building as a meaning-making activity—and how every patient and family is different. P2 noted that *professionals need to include the family and understand their demands, as individuals who possess diverse backgrounds and varying sentiments on the desire to reminisce*. Participants articulated the personal motivations of patients for establishing their legacy as *a desire for remembrance* (P6); *a quest for peace of mind, organizing emotional affairs, seizing the opportunity to document their thoughts and feelings, or a longing for a legacy from others* (P11); and *fostering a sense of connection for descendants, assuring them they are not isolated and are part of something greater than themselves* (P8). Furthermore, participants articulated how legacy activities or artifacts facilitated their understanding of their patient’s illness or death. P15 highlighted how the legacy item itself served as a tangible reminder for bereaved families of the purpose of a patient’s illness experience: *taking part of a grounding object of legacy allows families to ascribe meanings and comprehend the purpose of the illness, preparing them for loss*.

They also recognized legacy as an existential coping strategy defined by hope, life fulfillment, and acceptance of mortality. One participant said, *legacy is an anchor of hope for patients and families to help them have closure and would “lighten the load” near the EoL* (P10). For many patients, telling their loved ones about their accomplishments instills a sense of essence (continuity of self), and self-regard or pride. In the end, legacy is *a way to return to memories, reflect on them and validate their lives* (P9).

(b) Enduring connections in the context of death, dying and grief

Regardless of their scope, legacies were characterized as transcending the confines of life and death, embodying both a spiritual essence and an eternal spirit. At this point, P12 highlighted how legacy-based interventions *can reinforce spiritual connections between patients and their loved ones. After all, we are all spiritual beings who are part of something greater than ourselves.* Enduring connections refer to how individuals maintain ties, even after their passing, and to the ongoing impact of those relationships on the bereaved. Some participants shared their personal experiences, notably those related to loss and grief. P1 argued: *The creation of legacy items over time with patients and families facilitates their ongoing processes of loss and grief*. In parallel, P5 punctuated that legacies enable bereaved families to revisit their loved one’s memory and validate their existence: *They can touch and see their photos, digital stories, fingerprints…so it’s like they see them, right here*.

Intentional care through people’s participation in elements of care, extended family visitation, or making space for family private time or routines contributes to positive and dignified care in the last moments. One participant said: *Giving people time to be together, resolving issues, and resigning are ways to ensure a legacy* (P2).

Furthermore, some participants described how the experience of losing someone has helped families channel their grief into serving others, as a part of their life mission and a way to honor those who have gone before. P14 stated that *preserving a connection after the demise of a loved one is regarded as significant, and legacy remembrances may facilitate this process*. P16 also said, *I remember a family that created an association to support children with disabilities, after the death of their daughter, this was the tribute that the parents wanted to pay… to support other children*.

(c) Act of seeking validation for one’s existence

Several participants alluded to symbolic immortality through statements: *legacy is a way of perpetuating individual history* (P3), *the ability to immortalize existence* (P7) and even *the possibility of giving meaning to life* (P13). For some professionals (P6; P7), legacy initiatives (including hand molds, fingerprint charms, handprints, letters, pictures and art created by patients) provided concrete proof that a dying person once existed. Other participants, like P4 stated that: *some families want to create timeless keepsakes that perpetuate the most precious memories. More than an object, they need to live the last days with presence and unconditional love.*

Other types of legacy, such as positive memories, stories, relationships, and the impact the patient had on the lives of others, were referenced by participants as intangible legacies. Some study participants believed that individuals are integral to a profound heritage and sought to link past and future generations. P9 stated that EoL patients frequently desire to *instill a sense in their descendants that they are not isolated in the world and that they are connected to something far greater than themselves*. The process of introspection and contemplation confirmed people’s identity and life roles, affirming that they were not a failure. Also, other expressions of legacy may include letters as a nonlegal way to address mortality by communicating a lasting and intangible legacy of values to others. P8: *In fact, legacy letters offer individuals an opportunity to contemplate their lived experiences, the meanings acquired from them, and the importance of those experiences, thus transmitting a legacy of values to future generations*. This motivation emerged as a yearning for remembrance, recognition, and expression, as well as the articulation of thoughts sometimes omitted from a legal will.

#### 3.2.2. Enablers of Legacy-Building Process

Factors that facilitate legacy-making interventions include (a) building trust and effective communication, and (b) knowing personal stories.

(a) Building trust and effective communication

PC professionals acknowledged that fostering trust depended on their capacity to establish rapport through good communication. P1: *It is not possible to help someone at the end of their life except through the relationship that is established. It is the relationship that allows us to access the person’s story*. Other participants also recognized the need for comprehensive and transparent dialog throughout the legacy-building process. In this sense, P15 referred that *patients sought a detailed explanation of their options and a comprehensive understanding of the many forms of legacy, including the timing and recipients of item distribution.*

Empowering patients and advocating for them by granting control are essential in fostering trust with both the patient and their family, indicating a shared commitment to the patient’s well-being. Empowering a patient is essential, granting them control over the topics discussed with them and those shared with family or other selected individuals. P2 stated: *We often talk about tailoring plans to people, but this requires their active participation, based on their preferences. I believe this is how we preserve human integrity and dignity until the EoL. After all, people’s choices must be respected*. In this vein, P11 added *in the process of legacy building, the patient can exert autonomy and control by developing a legacy activity, potentially reducing some physical and mental burdens associated with the condition*. Ultimately, involving family members in the legacy creation process was proposed *to enhance possibilities for families to spend quality time together and engage in significant discussions* (P4).

(b) Knowing personal stories

Familiarity with the person and possessing a mutual understanding of their cultural background were perceived as enablers; however, self-awareness and reflexivity were equally deemed essential, recognizing that one’s own belief system may differ, irrespective of any assumed commonality, and avoiding preconceived notions when engaging with patients and families. Participants regarded a person-centered approach as essential for understanding and discerning their priorities. For P4, *professionals should be able to incorporate distinctive personal and familial characteristics into the legacy creation process to enhance the individuality and creativity of these initiatives*. P16 also recognized *creativity as an essential component of personalized care*.

Most participants underscored the significance of incorporating distinctive patient characteristics, personal recollections, or familial connections into the legacy-building process. On this point, P13 stated *the need to articulate specific traits, interests, or experiences of their patients as essential to the development of significant legacy goods*. P9 also said that *because young ill people are part of a digital generation, the user-friendliness of web-based legacy-making platforms [e.g., blogging* and social media] *could be a good option for them, which demands digital skills from professionals*. *This is a way for us to get closer to individual needs and characteristics*.

Two other participants reported that although patients’ physical condition worsened over time, being able to build their legacy was one of the few things that gave them *a sense of autonomy* (P3) and *independence* (P14). Consequently, it provided therapeutic advantages by fostering acceptance of their illness and grieving the loss of their future. Moreover, it provides a valuable opportunity for families to enjoy quality time together and engage in meaningful conversations with their loved ones.

#### 3.2.3. Challenges of Legacy-Building Process

Factors that hindered the legacy-making interventions included (a) lack of training, opportunities and time, (b) the emotional burden and (c) taboos surrounding death-related issues.

(a) Lack of training, opportunities and time

The prospects for engaging in legacy building were acknowledged as contingent upon a healthcare professional’s experience and confidence in addressing the topic. While participants acknowledged the significance of legacy-building strategies for patients and familial relationships, they expressed a need for further resources and training. They highlighted that they were not instructed on utilizing legacy promotion as a way for conserving dignity to improve family interactions. According to P12, *I only learned about legacy-promoting interventions during my master’s degree in PC. However, I feel unprepared to intervene and evaluate their implementation. This isn’t just my problem; other colleagues report the same thing*.

Time constraints and limited opportunities were also significant constraints identified by PC professionals when addressing the topic of legacy and any concerns from patients or families. Some professionals recollected instances when they failed to address legacies during EoL planning. P15 underscored: *when I began my professional practice in PC, I worked with a grandfather who wanted his unborn grandchildren to meet him. We tried to help him fulfil this wish, but the advanced stages of his illness prevented him from writing a legacy letter. While I recognized my inexperience, I missed an opportunity to address his wish*. P10 also expressed that *the legacy-building process should not be initiated late, as it runs the risk of being incomplete due to the patient’s worsening condition*. Moreover, the timing of the introduction of legacy activities was considered a crucial element of personalized care. The time of the explanation regarding legacy-building activities may significantly affect a person’s perceptions of the whole experience.

(b) Emotional burden

The unpredictable nature of some illnesses and the rapid decline in some cases can make it difficult to plan and execute legacy-building activities. The emotional burden of facing mortality and the absence of a shared future with the patients was also significant. This frequently resulted in feeling overwhelmed, eliciting intense emotional responses during construction of legacy items.

Clearly, legacy activities were highly sensitive to the passage of time and disease progression. As P9 suggested, *a decline in physical capabilities meant additional support was needed to build a legacy. I recall a patient I followed who was reluctant to record personal messages for his son when others were present. The patient struggled with the conflict between needing others present to help him with the equipment and not wanting them present during the recording*. Although initiating the intervention earlier in the disease progression was recommended, families were concerned that early engagement in legacy-making could diminish the patient’s prospect for survival.

Interestingly, two participants highlighted that emotional turmoil also affects professionals when they fear that their involvement in legacy-making might lead the ill person to lose hope for life. P16 reveals that, *even though I believe in the benefits of legacy promotion, my main concern is stealing hope.* On top of this, P7 also said *that talking about legacy can mean that we are abandoning the person and somewhat provoke some kind of suffering*.

(c) Taboos surrounding death-related issues

In an era of unprecedented openness and access to information, where everything is discussed, participants’ experience is that the societal taboo around death has worsened, and this often hinders the development of legacy-building activities. Besides, the current defensive medicine—a culture to always seek a cure—has colluded with this, the result being a disservice to the dying and their families. In this regard, P13 states *that helping someone die cannot be seen as a failure, but rather as an acknowledgment of human finitude… It is essential to inform patients and their families about the disease severity, so that they are aware of death and dying, and thus can prepare themselves… this is where legacy can be a valuable tool*.

In other cases, people struggle to accept a diagnosis and reveal unrealistic expectations about the future due to fear of death and unaddressed personal needs. At this point, P4 shared that *people often avoid discussing death-related issues, because they are afraid of death, and sometimes because they have lived personal stories of family death that were traumatic*.

Two other participants recognized that cultural and societal issues can also aggravate these taboos about death. P8 shared that *our culture and religion fail to educate people about death, increasing their fear of death.* Moreover, P10 points out *that planning for death is something that is difficult for most people to think or talk about. It’s enough to note that dying at home is a rarity today; many people at the end of their lives die in hospitals or nursing homes, far from their families. This increases fear and distances people from the most existential issues, such as legacy, which are healing for those who die and the bereaved.*

## 4. Discussion

This study contributes to understanding the experiences of PC professionals with legacy building as a dignity-conserving practice in EoL [28]. Our findings are aligned with previous literature indicating that the creation of a legacy allows individuals to confront mortality, gain self-awareness, experience personal growth and meaning, and bequeath a part of themselves to others, thereby fostering symbolic immortality [25,26]. Prior evidence indicates that a sense of personal control, particularly autonomy, can mitigate protective efforts to confront symbolic immortality. When an individual experiences choice and self-authorship, as when building a legacy, they contribute to their worldview and enhance a sense of symbolic transcendence, preparing them for death [46]. In Man’s Search for Meaning, Viktor Frankl highlighted that life’s significance can be discovered in every instant; existence perpetually retains meaning, even amidst suffering and death [47].

The theme “worth of legacy in EoL” identified in this study overlaps with the well-known construct of dignity in EoL, which highlights the importance of psychological, physical, and spiritual recognition for the dying patients and their family members [48]. Findings have shown that supporting patient dignity, and the holistic nature of the dying experience, requires interventions directed at promoting family dignity [49]. PC professionals in this study explained how important it is to consider family dignity in PC by considering their role as both a caregiver and a care receiver, and how promoting reciprocal and meaningful relationships facilitates the preparation for death and coping with grief [50].

Importantly, the findings of this study emphasize legacy as a process or experience rather than a tangible result; a more suitable designation for plaster legacy letters and other identical activities may be “mementos” or “keepsakes,” as they do not align with the legacy beliefs and wants of families and caregivers [51]. Providers should acknowledge that legacy encompasses the features, relationships, and shared experiences of the ill person and their family, rather than merely tangible things presented at or near EoL [25]. Evidence suggests that legacies can manifest in different forms, including biological legacy (e.g., genetics or health), material legacy (e.g., belongings or money), and a legacy of values (religion, culture, and tradition) [52,53]. For several people, the transmission of values is the paramount type of legacy bequeathal [54]. This discussion centers on a legacy of values or ethical wills as a way for transmitting intangible assets to subsequent generations (including emotional guidance and supportive instruction), rather than directives regarding the distribution of possessions or medical preferences at the EoL. The goal of establishing a legacy of values is to contemplate life’s experiences, to derive meaning, and to express values and wisdom as a constructive deed for one’s loved ones [35,55]. A recent scoping analysis of ethical wills, the most frequently referenced technique for documenting a legacy of values, revealed that individuals establish their legacy to confront mortality, promote generativity, reinforce identity, and rejuvenate intergenerational ties [35].

Notably, our findings indicate that effective communication, encompassing active listening, clear verbal communication, and mindful nonverbal communication in response to patients and families, can assist the PC team in understanding the essential relationships, characteristics, and experiences that can be integrated into the creation of keepsakes and genuinely legacy-focused interventions and support systems [25]. Besides, the authentic use of time, energy, and emotional support as families recount their narratives of life, love, and loss may serve as a viable and significant means to acknowledge and sustain the legacies of loved ones—an endeavor attainable within the framework of palliative and bereavement care [56]. Moreover, the platinum rule proposed by Chochinov [57], which advocates “doing unto patients as they would want done unto themselves”, may serve as a more suitable criterion for attaining optimal person-centered care. This entails understanding patients as individuals, therefore informing treatment choices and establishing a compassionate and respectful tone of care. It also encompasses a compassionate approach that fosters non-abandonment, brings hope, and aids personal growth and fulfillment [25].

Our study also revealed that PC professionals recognized concerns stemming from inadequate training, and a lack of opportunities for legacy-building interventions. Recent research indicated that healthcare professionals voice worries over lack of training and personnel shortages, resulting in delayed person-centered care [58,59], which should drive educational and healthcare institutions to develop dignity-in-care education [60]. Our findings also highlighted lack of time due to rapid decline of patient health that might significantly impact interpersonal relationships and dignity-supporting work environments [36]. The difficulties conveyed by the participants, in terms of emotional turmoil and taboos around death, induce feelings of dissatisfaction and might violate dignity in EoL care. Prior evidence demonstrated how dignity assurance in healthcare settings can be enhanced by adequate information, preserving patient autonomy and promoting socioemotional regulation [61]. Those taboos surrounding death are rooted in cultural beliefs that prevent people from expressing their preferences for care and create barriers to dignified EoL planning [62]. Addressing these taboos requires culturally sensitive communication training for healthcare providers and increased awareness of advanced care planning to ensure patient dignity is upheld.

### 4.1. Study Limitations

This is the first Portuguese study to explore the perspectives of PC professionals in creating legacy activities with patients and their families using in-depth interviews. The study’s design involved participants recruited from different teams, professions and seniority levels, presumably enhancing variability in response patterns. Additional strengths encompassed the utilization of a well-defined methodological framework accompanied by explicit theoretical assumptions, and the validation of findings through member checking with participants, thereby enhancing trustworthiness.

However, this study has several limitations. While there is some participant heterogeneity, the findings may not be transferable to professionals with diverse identifying features and experiences. Moreover, further studies should investigate the legacy-building process across various cultural backgrounds and professional expertise to comprehend how life experiences, the perceived intimacy of family or patients, and cultural context influence legacy creation. The purposive sampling technique employed presents a second shortcoming, as study participants may have been motivated to participate due to their interest in making legacy goods or engaging in other dignity-conserving actions. Another limitation might be that the interview questions were only pilot-tested with members of the research team without involving professionals from the PC practice field. In addition, no participant expressing negative viewpoints or experiences was identified, suggesting potential selection bias. While the study includes professionals from various areas and levels of clinical experience, we did not find differences between junior and senior staff. Ultimately, we could not prospectively assess the outcomes of establishing a legacy. Besides, the thematic analysis looks for commonalities of meaning and perspectives across people rather than understanding how a given person, in a given context, makes sense of a given phenomenon [44]. Thus, future research should triangulate the perspectives of PC professionals from different disciplinary backgrounds, patients, and families utilizing high-quality mixed methods approach.

### 4.2. Implications for Practice

The findings of this study may be applicable for PC professionals to develop legacy-based interventions that normalize life reflection and EoL preparation [63]. People frequently postpone EoL preparations until death is approaching or they obtain a terminal diagnosis and prognosis [64]. It is essential to assist patients in anticipating and preparing for the EoL while they are still able to plan, as many will address their existential inquiries in terms of living, and not just in terms of dying [65,66]. The establishment of a legacy prior to death aids individuals in recognizing opportunities for transformation or ongoing development, reinforcing meaning, and strategizing for intentional living in their remaining time [66]. Future research should investigate how legacy creation can enhance potential selves and facilitate a dignified death among PC patients.

Healthcare providers should select suitable spiritual care interventions for patients based on their spiritual requirements, preferences, and traditions [67]. Subsequently, a suitable strategy must be devised based on the patients’ health status and a daily timetable to ensure the thoroughness of the intervention. Creating legacy-building interventions can assist interdisciplinary team members in engaging patients to reflect on their priorities when they initiate or revise EoL planning. These findings enrich the literature by providing insights into promoting deliberate living and sustained life involvement among PC patients and their families. Moreover, they reinforce the importance of establishing online platforms for families to exchange their legacy-making experiences, along with developing assessment criteria to measure the immediate and prolonged effects of these activities on PC patients, their families, and healthcare professionals.

Education on death, dying, and dignity-conserving EoL care must be essential, comprehensive, and mandatory in the curriculum for all health and social care students, as well as in the continuing education of practicing professionals. At a social level, the global movement of compassionate communities can foster legacy activities by normalizing conversations around death and dying, encouraging civic participation in EoL care, and providing social solidarity for individuals and families facing these experiences [68,69]. Ultimately, by giving a greater grasp of legacy construction, our findings may help healthcare providers better understand how to provide dying patients and their families with dignity-conserving care.

## 5. Conclusions

This study highlights the perspectives of PC professionals regarding the legacy-building process in PC settings, which potentially benefits PC patients and their families in preparing for EoL situations, fostering hope, gratitude, and peace. Our findings highlight legacy as a process or experience rather than a product, which means that establishing a legacy enables individuals to create a lasting impact on others, convey their core values, achieve a sense of tranquility, and promote ongoing involvement in life. Establishing a legacy may support people facing serious illness and their families to acknowledge their past, reframe their lives, and contemplate how they wish to perpetuate the legacy they have already formed through self-transcendence. Building trust and effective communication, and knowing personal stories, were identified as enabling legacy-making interventions. Challenges related to taboos surrounding death, emotional turmoil inherent to the decline of patient health, and professional issues (such as insufficient training, time and opportunities to apply legacy making interventions) were found to hinder the quality of care provided. By integrating these findings into clinical practice, potential benefits could be guided by future person-centered holistic interventions that assist palliative patients in preparing for the future while engaging in the present, contemplating their goals and decisions, improving communication with others, and fostering the next generation through the creation and dissemination of their legacies.

## Figures and Tables

**Table 1:** Background of participants (N = 16).

Participants	Gender	Age	Profession	Work Context	Length of Professional Experience in PC
P1	Female	42	Nurse	Palliative home-based team	5 years
P2	Female	33	Nurse	Inpatient PC unit	9 years
P3	Female	48	Physician	Inpatient PC unit	12 years
P4	Male	59	Physician	Inpatient PC unit	15 years
P5	Female	32	Nurse	Intra-hospital PC support team	1 year
P6	Female	47	Psychologist	Intra-hospital PC support team	3 years
P7	Female	46	Physician	Palliative home-based team	7 years
P8	Male	31	Nurse	Intra-hospital PC support team	2 years
P9	Female	55	Psychologist	Palliative home-based team	3 years
P10	Female	49	Nurse	Intra-hospital PC support team	7 years
P11	Female	43	Nurse	Palliative home-based team	5 years
P12	Female	44	Nurse	Palliative home-based team	7 years
P13	Male	47	Physician	Intra-hospital PC support team	8 years
P14	Male	31	Nurse	Inpatient PC unit	4 years
P15	Female	39	Nurse	Inpatient PC unit	10 years
P16	Male	28	Nurse	Intra-hospital PC support team	1 year

**Table 2:** An overview of the themes and subthemes.

Themes	Subthemes
The worth of legacy in EoL	(a) Process of memory-making (Memorabilia); (b) Enduring connection in the context of death, dying and grief; (c) Seeking validation for one’s existence
Enablers of legacy-building process	(a) Building trust and effective communication; (b) Knowing personal stories.
Challenges of legacy-building process	(a) Lack of training, opportunities and time; (b) Emotional burden; (c) Taboos surrounding death-related issues.

## Data Availability

The raw data supporting the conclusions of this article will be made available by the authors on request.

## Supplementary Materials

The following supporting information can be downloaded at: https://www.mdpi.com/article/10.3390/nursrep15100366/s1, Standards for Reporting Qualitative Research (SRQR) checklist.

## Public Involvement Statement

No public involvement in any aspect of this research.

## Guidelines and Standards Statement

This manuscript was drafted against the SRQR (Standards for Reporting Qualitative Research) [39] for qualitative research.

## Abbreviations

The following abbreviations are used in this manuscript:

EoL	End of Life
PC	Palliative Care

## Appendix A. Interview Guide

Participant description—gender, age, profession, work context, specific training in PC and length of professional experience in PC

Meanings Attributed to the construction of the EoL legacy

1. What does legacy mean to you? How do you define it?

2. What are the main attributes (characterizing elements) of the EoL legacy?

Experiences in the construction of the EoL legacy

3. Can you tell me about your last or most significant legacy-building experience? How would you describe it?

4. What strategies can be employed to foster legacy building with the person at the EoL and their family?

5. What benefits, in your view, arise from legacy building…

… for the person at the EoL?

… for family members?

… for professionals?

Facilitators and Obstacles to Legacy Building

6. What enablers do you face in implementing legacy-making interventions? (individual, relational, and organizational spheres)?

7. What are the barriers do you face in implementing legacy-making interventions? (individual, relational, and organizational spheres)?

7.1. How do these challenges, as you experience them, influence the quality of care you provide?

8. What would you need to do to better support these individuals and their families in building their legacy?

Conclusion

9. Is there anything we haven’t covered yet that you consider relevant?
